# An Atypical Radiologic Presentation of Right Sixth Lateral Rectus Palsy: A Case Report

**DOI:** 10.7759/cureus.32597

**Published:** 2022-12-16

**Authors:** Anicia Mirchandani, Sheena Saleem, Lalitha Sivaswamy

**Affiliations:** 1 Radiology, Detroit Medical Center/Wayne State University, Detroit, USA; 2 Pediatric Imaging, Children's Hospital of Michigan/Wayne State University, Detroit, USA; 3 Neurology, Children's Hospital of Michigan/Wayne State University, Detroit, USA

**Keywords:** horizontal diplopia, neuroradiology, wallerian degeneration, abducen nerve palsy, cranial nerve 6 palsy

## Abstract

Isolated ocular palsies are often associated with a benign process in the pediatric population but early diagnosis is critical to exclude any serious pathology. In this case, a six-year-old female with no significant past medical history presented with isolated right-eye medial deviation. The patient reported right-eye medial deviation for the past several weeks and associated double vision, but denied any pain with eye movements, other cranial nerve changes, or headaches. This case highlights the key radiologic finding which may ultimately allow for a leading diagnosis and inform further management in cases of isolated ocular nerve palsy.

## Introduction

Isolated sixth nerve palsy is the most commonly seen cranial nerve palsy in the pediatric population [1]. It necessitates a thorough investigation including imaging workup given its potentially serious common causes such as intracranial mass, elevated intracranial pressure, or stroke [2]. Isolated cranial nerve palsies are in fact one of the more common indications for neuroimaging [3], and MRI has been shown to detect lesions and conditions which may have otherwise gone undetected [4]. This case demonstrates the critical role of MRI by highlighting a case with unique radiologic findings consistent with Wallerian degeneration which has rarely been reported in the context of cranial nerve injury.

## Case presentation

A six-year-old girl presented to the neurologist with a history of strabismus of new onset. She reported right-eye medial adduction for the past several weeks and associated double vision. She was able to realign her vision by readjusting her gaze. She denied other cranial nerve symptoms, changes in cognition, or headaches. Family, social, and birth history were non-contributory.

**Figure 1:**
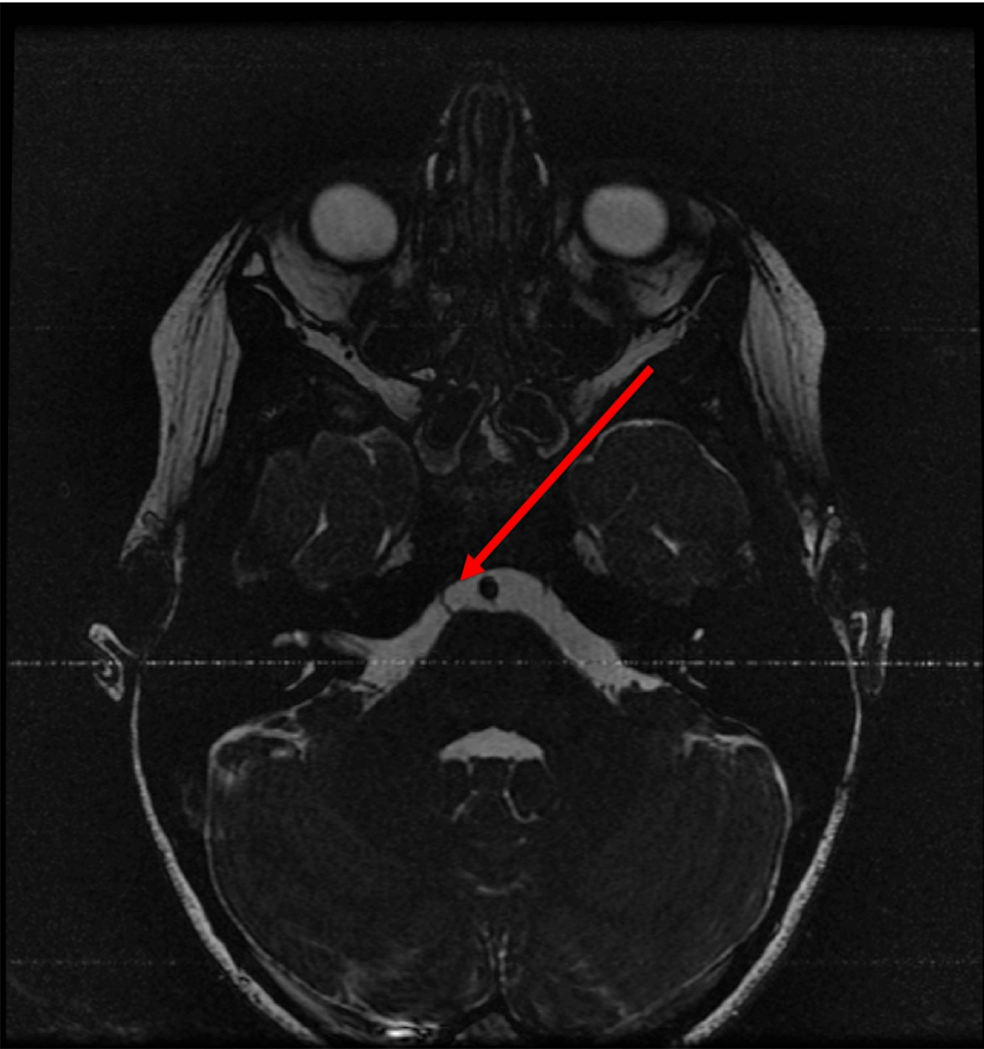
Fast imaging employing steady-state acquisition (FIESTA) sequence axial image delineates the course of the right sixth cranial nerve.

**Figure 2:**
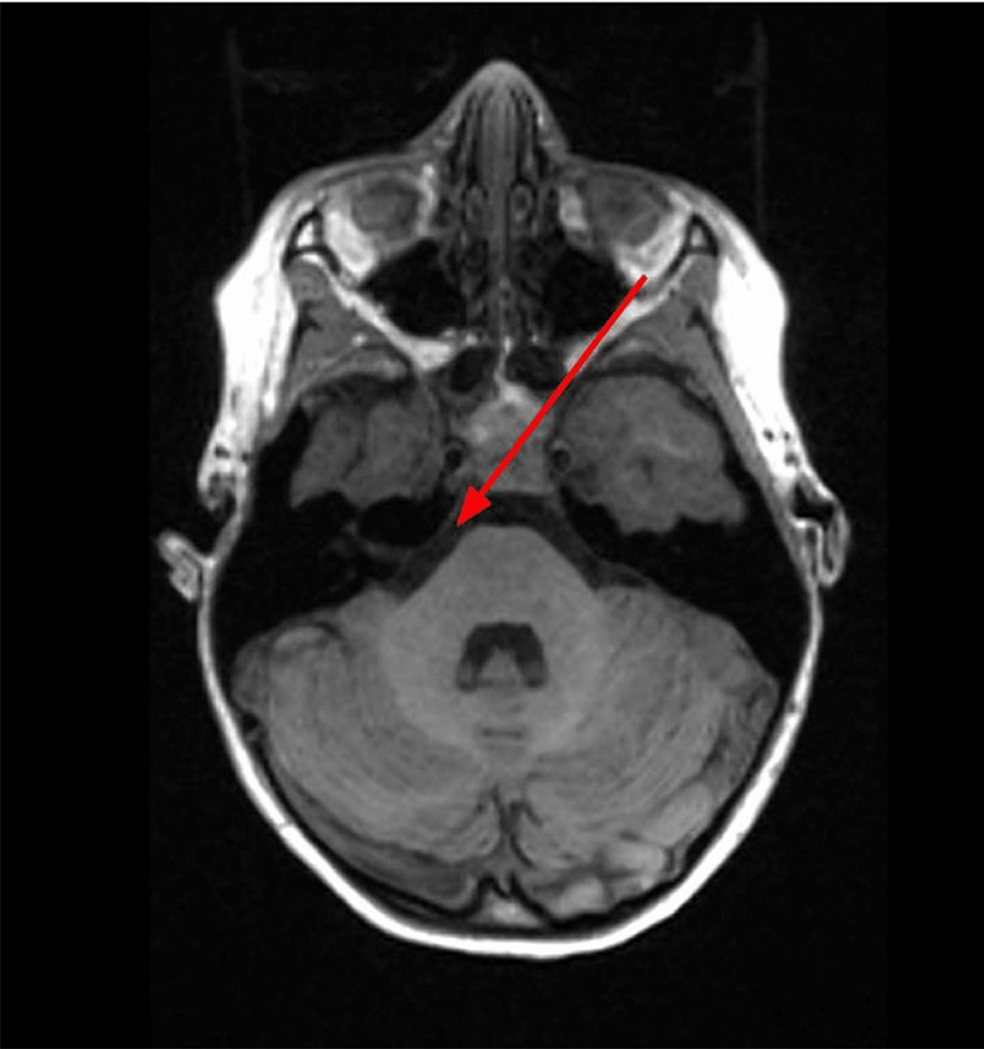
T1 axial demonstrating no significant hyperintensity of the right sixth cranial nerve.

**Figure 3:**
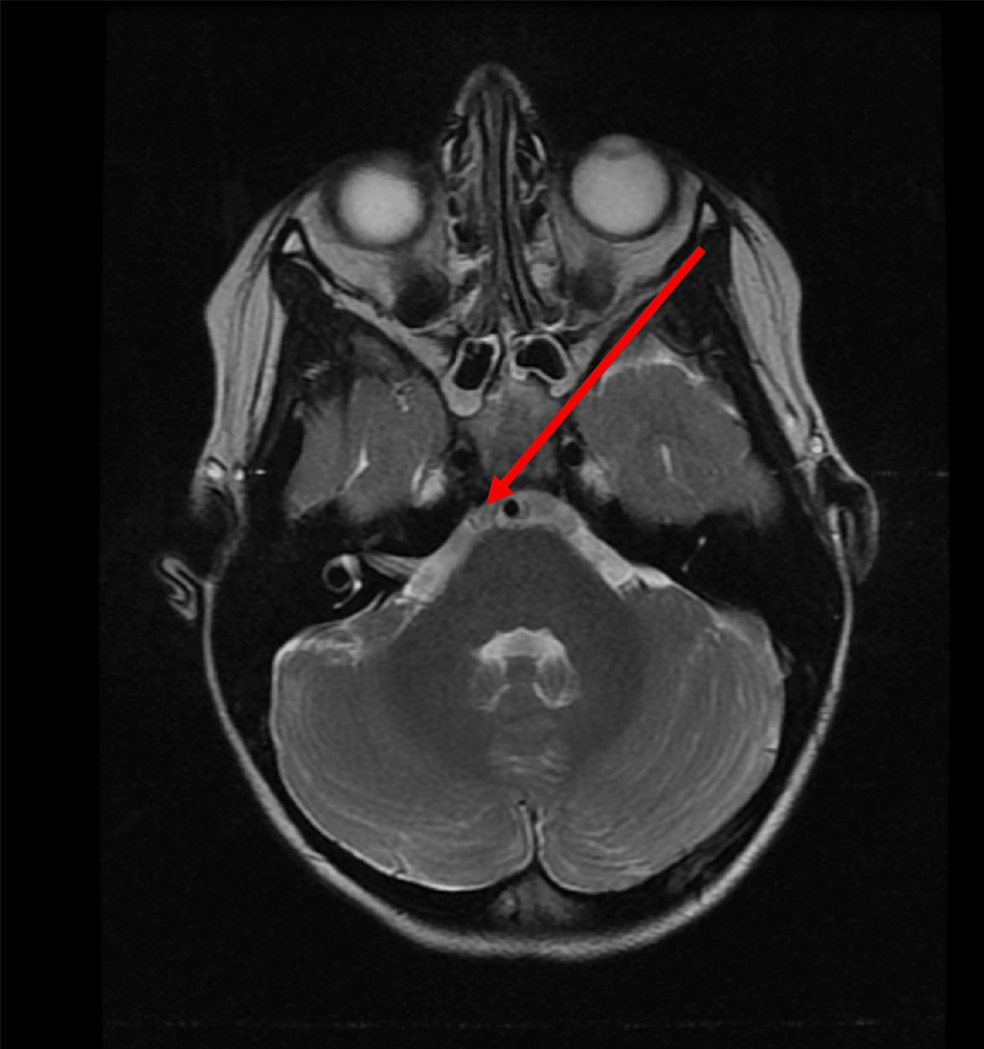
T2 axial demonstrating no significant hyperintensity of the right sixth cranial nerve.

**Figure 4:**
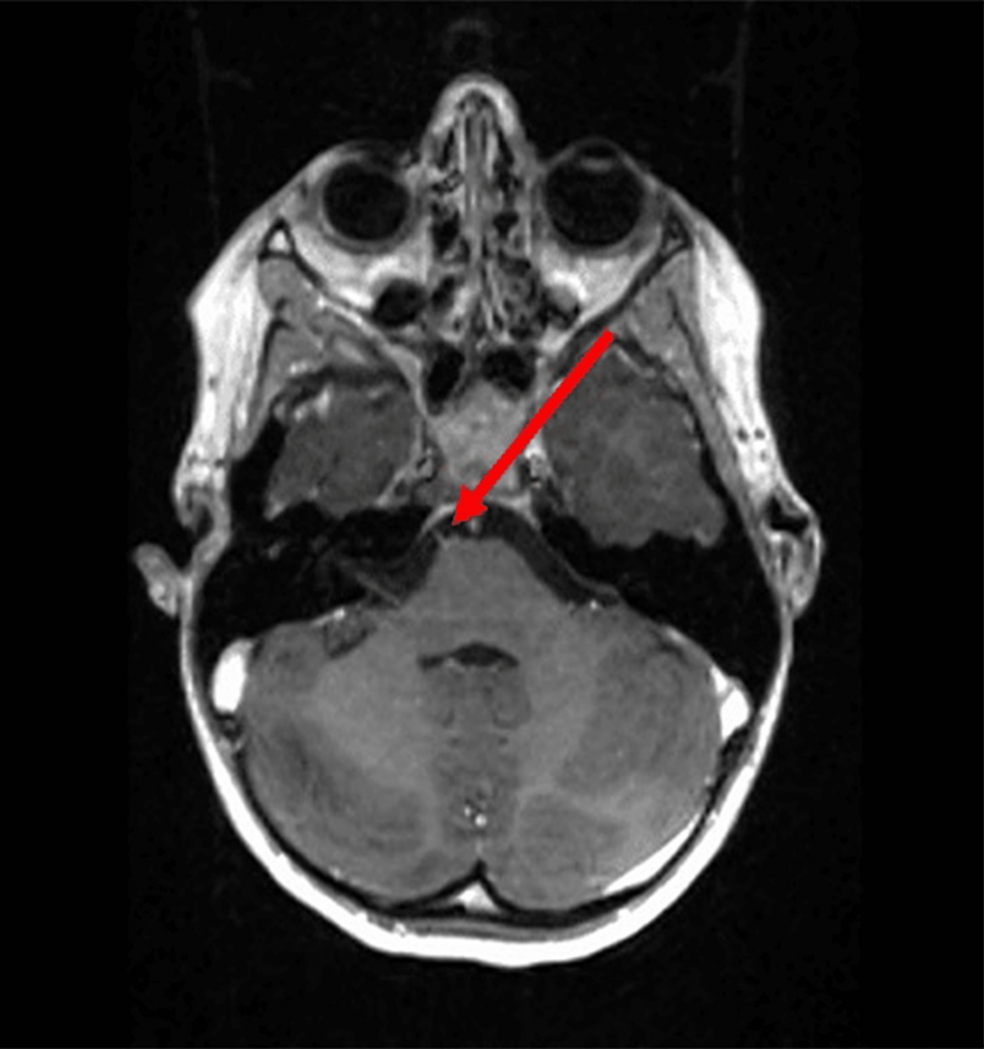
Enhancement of the right sixth cranial nerve on axial post-contrast spoiled gradient recalled echo (SPGR) sequence.

On neurologic exam, pupils were equal, round, and reactive to light and cranial nerves were intact with the exception of the failure of abduction of the right eye. Vitals and labs were also obtained and were all within normal limits including blood pressure and complete blood count, respectively. The MRI of the brain with and without contrast was then ordered to investigate potential etiologies and help determine the need for additional workup. Multiple sequences were obtained including T1, T2, fast imaging employing steady-state (FIESTA) sequence, and post-contrast spoiled gradient recalled echo (SPGR) sequence. FIESTA sequence helped to clearly delineate the course of the right sixth cranial nerve (Figure 1). In the T1 sequence (Figure 2) and T2 sequence (Figure 3), the right sixth cranial nerve is not clearly visible and there is no significant hyperintensity. On post-contrast SPGR sequence, there was demonstrated medial adduction of the right globe and asymmetric enhancement of the right sixth cranial nerve which appeared symmetric in size to the left sixth cranial nerve (Figure 4). 

## Discussion

Initially, in terms of isolated right ocular nerve palsy, there is an immediate concern for an intracranial neoplastic process, prompting the need for further imaging. The differential considerations for sixth cranial nerve palsy can be thought of in terms of the course of the ocular nerve and involvement of contiguous structures and can be broadly thought of in terms of six categories, namely brainstem syndrome, elevated intracranial pressure syndrome, petrous apex syndrome, cavernous sinus syndrome, orbital syndrome, and isolated sixth nerve palsy syndrome [5].

In brainstem syndrome, a lesion in the posterior fossa is expected to involve the fifth, seventh, and eighth cranial nerves, as well as the sixth nerve, which would present with additional clinical symptoms such as ipsilateral Horner’s syndrome (Foville’s syndrome) or contralateral hemiparesis (Raymond’s syndrome) [6]. In elevated intracranial pressure syndrome, there may be associated radiologic findings of normal or small-sized ventricles (not seen in this case) and downward displacement of the brainstem leading to stretching of the sixth cranial nerve in the subarachnoid space, which is susceptible to increased pressures due to its long course [7].

In petrous apex syndrome, the tip of the petrous pyramid makes the portion of the sixth nerve in Dorello’s canal more prone to pathologic processes. A well-described phenomenon is Gradenigo syndrome, with symptoms of facial pain (trigeminal neuropathy) and otomastoiditis, in addition to lateral rectus palsy [8] (symptoms also not described in our patient). In cavernous sinus syndrome, the course of the sixth nerve is associated with the internal carotid artery, carotid sympathetic plexus, and third, fourth, and fifth nerves, and would likely consist of clinical defects of at least two or more of these structures [9]. In the orbital syndrome of sixth nerve palsy, we would expect to see involvement of the optic nerve, which may present as papilledema or optic atrophy [5]. Lastly, in isolated sixth nerve palsy syndrome, as seen in our case, it is often attributed to vascular factors such as diabetes, hypertension, migraine presentation, or immunologic damage, which can affect the nerve anywhere along its length [10].

**Figure 5:**
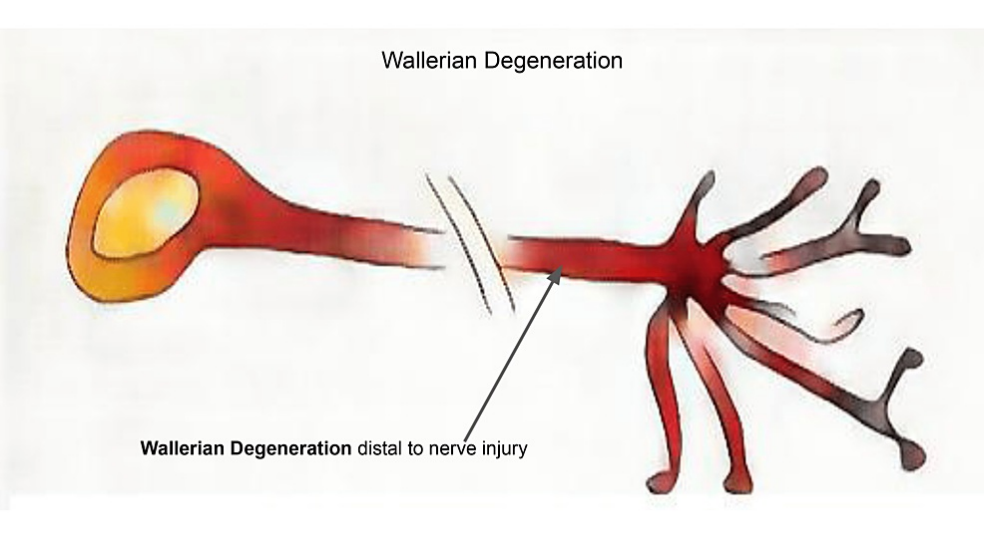
Illustration of Wallerian degeneration of axon and myelin sheath distal to proximal nerve injury Image created by the author, Dr. Mirchandani.

Given that our patient’s presentation was most consistent with isolated lateral palsy syndrome and the clinical history did not point to a specific vascular or predisposing factor, an immunologic basis was favored. The patient’s isolated palsy and enhancement characteristics are consistent with Wallerian degeneration of the right sixth nerve, which is an innate immune response to remote nerve injury [11]. In Wallerian degeneration, the nerve fibre axons are damaged proximally, and the axon and its accompanying myelin sheath then undergo an anterograde degeneration process (Figure 5) [12].

Radiologically, Wallerian degeneration can be further classified into four stages by timeline. Stage 1 has been described as the degeneration of axons and myelin sheaths with mild chemical changes and no significant T1/T2 signal changes (zero to four weeks). Stage 2 has been described as the rapid destruction of myelin protein fragments that were already degenerated, with the lipid component remaining intact (four to 14 weeks) and corresponding T1 hyperintensity and T2 hypointensity. Stage 3 is when the degenerated axons and myelin sheaths are replaced by gliosis (>14 weeks) with associated T1 hypointensity and T2 hyperintensity. Stage 4 is brainstem atrophy with or without hypointensity [13]. This patient’s presentation is most suggestive of Stage 2 Wallerian degeneration (four to 14 weeks), which involves rapid destruction of myelin protein fragments with an intact lipid component and corresponding T1 hyperintensity and T2 hypointensity. In our study, this was best demonstrated by the post-contrast SPGR sequence, resulting primarily in T1 or proton density contrast by semi-randomly changing the phase of the radiofrequency pulse and spoiling the transverse steady state [14].

## Conclusions

Isolated cranial nerve palsy in the pediatric population may represent a benign versus serious pathologic process, the determination of which can significantly impact patient management. This case uniquely demonstrates Wallerian degeneration in terms of an isolated cranial nerve injury and the image-specific findings that ultimately led to the key diagnosis and further management for this patient, which involved strabismus surgery with the expectation of partial recovery.
